# Pheromone binding proteins enhance the sensitivity of olfactory receptors to sex pheromones in *Chilo suppressalis*

**DOI:** 10.1038/srep13093

**Published:** 2015-08-27

**Authors:** Hetan Chang, Yang Liu, Ting Yang, Paolo Pelosi, Shuanglin Dong, Guirong Wang

**Affiliations:** 1 State Key Laboratory for Biology of Plant Diseases and Insect Pests, Institute of Plant Protection, Chinese Academy of Agricultural Sciences, Beijing, 100193, China; 2 Education Ministry, Key Laboratory of Integrated Management of Crop Diseases and Pests, College of Plant Protection, Nanjing Agricultural, Nanjing, 210095, China

## Abstract

Sexual communication in moths offers a simplified scenario to model and investigate insect sensory perception. Both PBPs (pheromone-binding proteins) and PRs (pheromone receptors) are involved in the detection of sex pheromones, but the interplay between them still remains largely unknown. In this study, we have measured the binding affinities of the four recombinant PBPs of *Chilo suppressalis* (CsupPBPs) to pheromone components and analogs and characterized the six PRs using the *Xenopus* oocytes expression system. Interestingly, when the responses of PRs were recorded in the presence of PBPs, we measured in several combinations a dramatic increase in signals as well as in sensitivity of such combined systems. Furthermore, the discrimination ability of appropriate combinations of PRs and PBPs was improved compared with the performance of PBPs or PRs alone. Besides further supporting a role of PBPs in the pheromone detection and discrimination, our data shows for the first time that appropriate combinations of PRs and PBPs improved the discrimination ability of PBPs or PRs alone. The variety of responses measured with different pairing of PBPs and PRs indicates the complexity of the olfaction system, which, even for the relatively simple task of detecting sex pheromones, utilises a highly sophisticated combinatorial approach.

Insects monitor the chemical environment with specialised chemosensilla localised on dedicated sensory organs, such as antennae and mouth parts, but also on other parts of the body^1^. Olfactory and gustatory sensilla contain the dendrites of sensory neurons, expressing on their membranes specific chemoreceptor proteins^2^,^3^,^4^. These olfactory (ORs) and gustatory receptors (GRs) span the dendritic membrane with seven a-helices, but are not G-coupled receptors and present an inverted topology with their C-terminus in the extracellular space^5^. Moreover, insect ORs are heterodimeric complexes with a constant member named ORCO (Olfactory receptor coreceptor)^6^,^7^. Such heterodimers are responsible for detecting and discriminating odorants and pheromones in insects.

The space between the dendritic membrane and the cuticular wall is filled with soluble proteins with affinity for small ligands, present at concentrations in the millimolar range. These proteins belong to two different families, OBPs (odorant-binding proteins) and CSPs (chemosensory proteins), each including several members with different spectra of affinities to pheromones and odorants^8^,^9^,^10^,^11^. Recent research suggests that OBPs are required for a correct detection of semiochemicals. Silencing OBP genes abolished or modified behavioural and electrophysiological responses to odours and pheromones in several species^12^,^13^,^14^. In other cases, binding assays and behaviour experiments have indicated that specific OBPs mediate detection and discrimination between different semiochemicals^15^,^16^,^17^.

Insect OBPs are folded into a very compact and conserved structure made of six a-helices enveloping a hydrophobic binding pocket^18^,^19^. Six cysteines paired in three disulphide bridges greatly contribute to the exceptional stability of these proteins^20^,^21^. Within the OBPs of Lepidoptera a sub-class can be identified on the basis of sequence homology encoding those members tuned to sex pheromones. The assignment is robust and reliable, being based on several pieces of evidence. The first insect OBP to be discovered binds the sex pheromone of the giant moth *Antheraea polyphemus*^8^ and therefore was named PBP (pheromone-binding protein). It has also been shown that in several species sensilla trichodea, specifically tuned to sex pheromones, express PBPs^22^,^23^,^24^. Moreover, in some cases coexpression of olfactory receptors responding to pheromones and PBPs has been visualised in the same sensillum^25^,^26^.

Thanks to new generation rapid and economical methods of sequencing, the complete repertoire of ORs and OBPs for a large number of insect species is available in the databases. As observed for the family of OBPs, also within the large repertoire of ORs a subset of few members can be identified in Lepidoptera on the basis of sequence analysis as putative receptors for sex pheromones (PRs: pheromone receptors). Such assumption is mainly based on the identification of the first lepidopteran member of this sub-group in *Bombyx mori*^27^. This receptor is specifically expressed in male antenna, in dedicated trichoid chemosensilla responding to the sex pheromone bombykol. The same sensilla also contain PBP1. Moreover, this receptor responds to bombykol when expressed in *Xenopus* oocytes^28^. In several moth species a small number of ORs bear significant similarity to *B. mori* OR1 and are therefore confidently assumed to be tuned on sex pheromones. In some cases such assumption has been validated by functional studies with ORs expressed in heterologous systems^26^,^29^,^30^,^31^.

Using the robust system of *Xenopus* oocytes to functionally characterise ORs, recent studies have addressed the question on the role of OBPs by adding this proteins to the test and recording responses to pheromones in their presence. In experiments of this type, performed with the PRs of *B. mori*, *Antheraea polyphemus* and the diamond back moth *Plutella xylostella*, a stronger and more specific electrophysiological response was measured when the pheromone was applied together with the appropriate PBP^26^,^32^,^33^. However, in a single report an opposite behaviour was observed explained with a trapping effect of the PBP, thus resulting in reduced responses^34^.

While a requirement of OBPs in insect olfaction has been clearly demonstrated, their mode of action and in particular their interactions with the corresponsing receptors is still a matter of debate. Models have been proposed, where the binding of pheromones to the correspinding PBPs induces conformational changes in the protein, that in some way leads to activation of the receptor. The mechanism first described in *Bombyx mori* PBP1 and then reported for several other OBPs involves the C-terminus of the protein, which undergoes a drastic conformational change with pH. Around neutrality the C-terminus fragment is not structured and interacts with the solvent, while the pheromone occupies the binding site. At low pH values, as those occurring close to the membrane and therefore in the proximity of olfactory receptors, the C-termins folds into a seventh a-helical domain, which enters the binding pocket, thus pushing out the bound pheromone and presenting it to the receptor^35^,^36^. Although the conformational change has been clearly demonstrated at the structural level through X-ray diffraction and NMR studies, the significance of such model in the activation of the receptor has been questioned^37^.

In any case, this phenomenon can only occur in OBPs with a C-terminus long enough to produce an additional helix. In other, shorter, OBPs the last few amino acids can act as a lid to cover the binding pocket, while in those with the shortest C-terminus the binding site remains always open to the environment. A classification of these proteins into long, medium and short OBPs has been suggested based on such considerations^19^.

Whatever the mechanism of interplay between OR, OBP and odorant, it is generally accepted that the first two components are both required for a correct detection of the stimulus. Therefore, a functional study of ORs should better be performed in the presence of OBPs, in order to reproduce *in vitro* a system as similar as possible to the physiological conditions. This approach requires first the matching OBP for each OR to be identified. This is far from easy task, given the high numbers (in the range of a couple of dozens) of both ORs and OBPs in most insect species so far studied, producing a number of theoretical combinations in the order of several hundreds to few thousands. Besides, in some instances cooperation between two OBPs, expressed in the same sensillum, has been observed^38^,^39^.

Given these difficulties, the sub-system present in Lepidoptera, constituted by PRs and PBPs and dedicated to the perception of sex pheromones, offers a simplified scenario to model and investigate interactions between ORs and OBPs. This sub-group of proteins generally contains 3–4 members for each family, making functional studies feasible and better focused. Moreover, both PRs and PBPs are easily and confidently discriminated from other ORs and OBPs, respectively, on the sole basis of sequence comparison^40^,^41^.

Comparing the responses of ORs to a series of ligands in the presence and in the absence of OBPs and bringing in the same picture the binding affinities of OBPs to the same ligands can eventually throw some light on the interplay between these three chemical performers and suggest experiments to investigate their interactions in the chemosensillum.

Finally, understanding the role of OBPs in odour recognition, and specifically of PBPs in pheromone detection, may suggest focused strategies to disrupt semiochemical detection and recognition in agricultural pests. In fact OBPs represent more approachable targets with respect to ORs, being small, soluble, stable and easier to manipulate and modify.

The striped rice stemborer *Chilo suppressalis*, Walker (Lepidoptera: Pyralidae) is responsible for great losses in agriculture, particularly in Asian countries. It feeds on many monocotyledons and dicotyledons and is a major pest of rice. The sex pheromone of *C. suppressalis* is a blend of (*Z*)-11-hexadecenal (Z11–16:Ald), (*Z*)-9-hexadecenal (Z9–16:Ald) and (*Z*)-13-octadecenal (Z13–18:Ald), with the first compound accounting for around 80% and the other two in about equal parts for the remaining 20%. In the field, a blend of the three components is effective in trapping males^42^,^43^.

A transcriptome project on the antennae of *C. suppressalis*^44^ revealed the presence of four genes encoding proteins of the PBP sub-family and six encoding PRs. In this study, we have measured the binding affinities of the four PBPs expressed in a bacterial system to pheromone components and structurally related compounds. We have also expressed the six PRs in *Xenopus* oocytes and monitored their responses to the same ligands in the presence of each PBP by voltage-clamp electrophysiological recordings.

## Results

### Tissue expression profiles of *CsupPRs* and *CsupPBPs*

**Figure 1:**
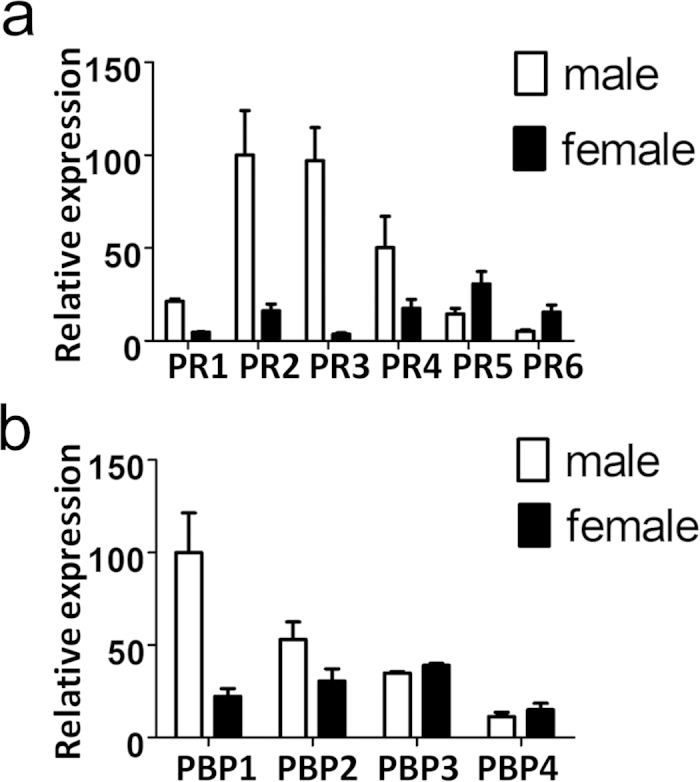
Relative expression of *CsupPRs and CsupPBPs* in antennae of adults. The expression levels are calculated relative to that of the housekeeping gene, *CsupG3PDH* and normalised on the values of male *CsupPR2* and *CsupPBP1* set to 100. Experiments were performed in triplicates. Error bars indicate SEM.

In order to investigate the functional relationships between the six PRs and the four PBPs in the complex process of detecting and discriminating the specific sex pheromones in *C. suppressalis*, we first mapped the expression of their genes in antennae and other parts of the body of adult females. As expected, we found all the PRs and the PBPs to be present almost exclusively in the antennae (results not shown). Quantitative PCR, performed in duplicates on male and female antennae (Fig. 1) revealed the relative levels of expression for both PRs and PBPs, perhaps reflecting relative importance or environmental abundance of their relative ligands^45^. In particular, we observe that genes encoding PR1 to PR4 are much more expressed in males than in females, while the situation is reversed for PR5 and PR6.

### Ligand-binding affinities of CsupOBPs

**Figure 2:**
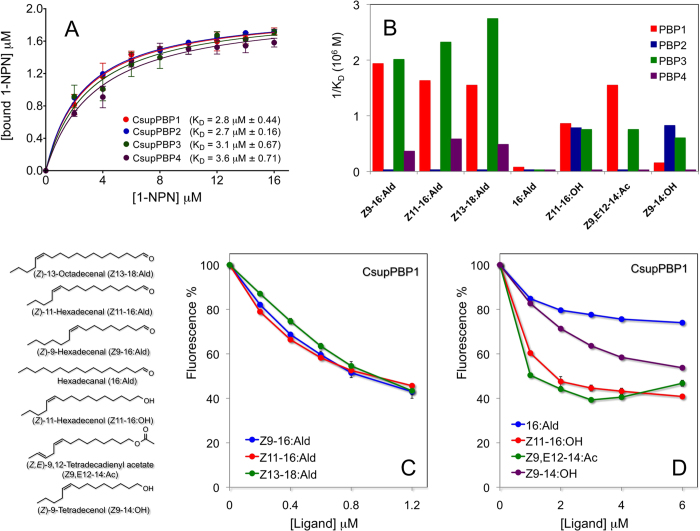
Ligand binding assay of CsupPBPs. (**a**) Binding curves of 1-NPN to CsupPBPs (**A**) and competitive binding curves of CsupPBP1 to sex pheromone components (**C**) and analogs (**D**). Displacement curves for CsupPBP2, CsupPBP3 and CsupPBP4 are reported in Figure S1. Experiments with 1-NPN and with competitors were performed in triplicates and mean values and standard errors are reported. The Prism software was used to analyse the data and plot the binding curves Panel (**B**) reports in graphical form the affinities of the four PBPs to the seven ligands. For a more immediate visualisation, the reciprocal of dissociation constants have been plotted. Structures of the ligands utilised are also reported in the same figure.

To probe the affinities of the PBPs for their ligands, we performed binding experiments, using the fluorescent displacement assay (Fig. 2). As ligands, we used the three pheromone components, all linear monounsaturated aldehydes. We also included four structurally related compounds comprising linear aldehydes, alcohols and acetates of 14 and 16 carbon atoms The four PBPs all exhibit good affinity to the fluorescent probe 1-NPN with similar dissociation constants around 3 µM (Panel A). They also bind all three pheromone components with some marked differences: PBP1 and PBP3 showed much better affinities to all three components than PBP2 and PBP4. Panels C and D in the same figure report the competitive displacement curves relative to PBP1 as an example, while the dissociation constants relative to all four PBPs are graphically illustrated as their reciprocals for more immediate comparison in panel B. The competition curves relative to the other three PBPs are reported in Figure S1. All experiments were performed in triplicates and average values were plotted together with relative SEM.

### Responses of PRs to pheromone components *in vitro*

**Figure 3:**
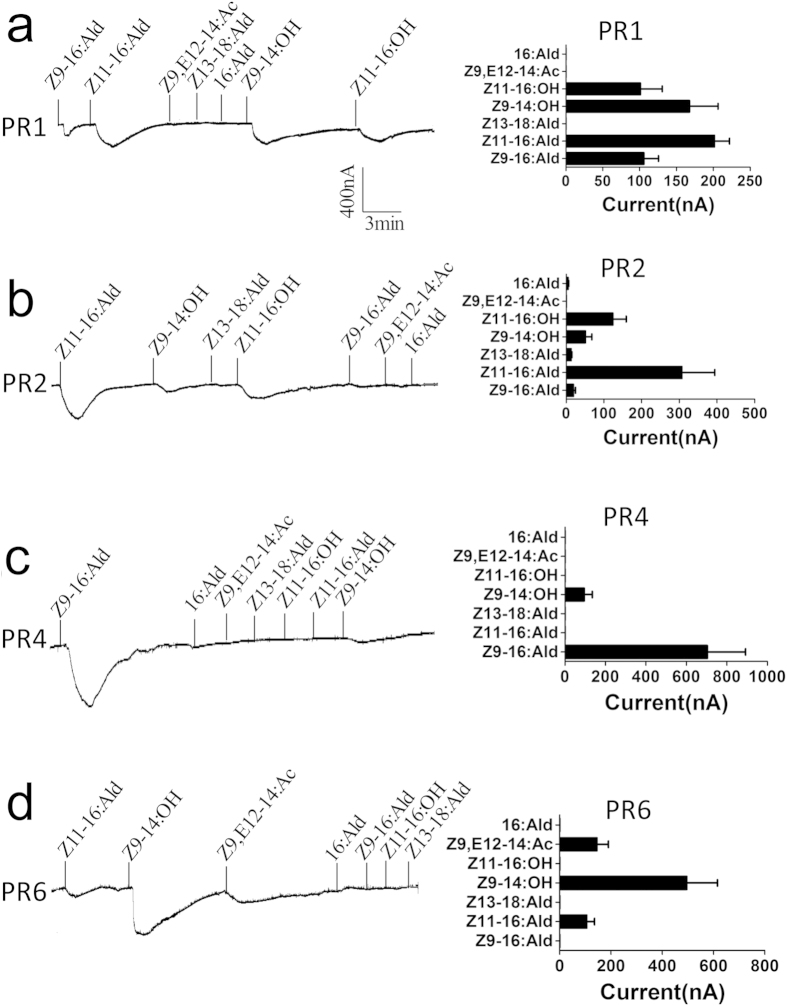
Functional analysis of *CsupPR* genes in *Xenopus* oocytes. In each panel: (*Left*) Inward current responses of CsupPR/CsupOrco-coexpressed *Xenopus* oocytes to 10^−4^ mol/L sex pheromone components and analogs. (*Right*) Response profiles of CsupPRs. Error bars indicate SEM (n = 6).

The affinities of PRs for their potential ligands were assayed by voltage clamp recording from *Xenopus* oocytes, which had been transformed with the genes encoding both Orco and the PR of interest. Responses were recorded after stimulation with the three pheromone components (Z11–16:Ald, Z9–16:Ald and Z13–18:Ald) and four analogues (Z9–14:OH, Z11–16:OH, 16:Ald and Z9,E12–14:Ac) all used at the same concentration of 0.1 mM. Figure 3 reports for each PR an example of the actual traces and the averaged values obtained in six replications. Four PRs produced strong signals when stimulated with the seven compounds, exhibiting different spectra of response, while PR3 and PR5 did not respond to any of the chemicals tested. PR1 showed the poorest specificity, responding to four of the seven compounds with signals of similar intensity, while PR4 proved to be specifically tuned to one of the two minor pheromone component Z9–16-Ald. PR2 and PR6 showed an intermediate behaviour with some selectivity.

### PBPs enhance the responses of PRs to pheromones

**Figure 4:**
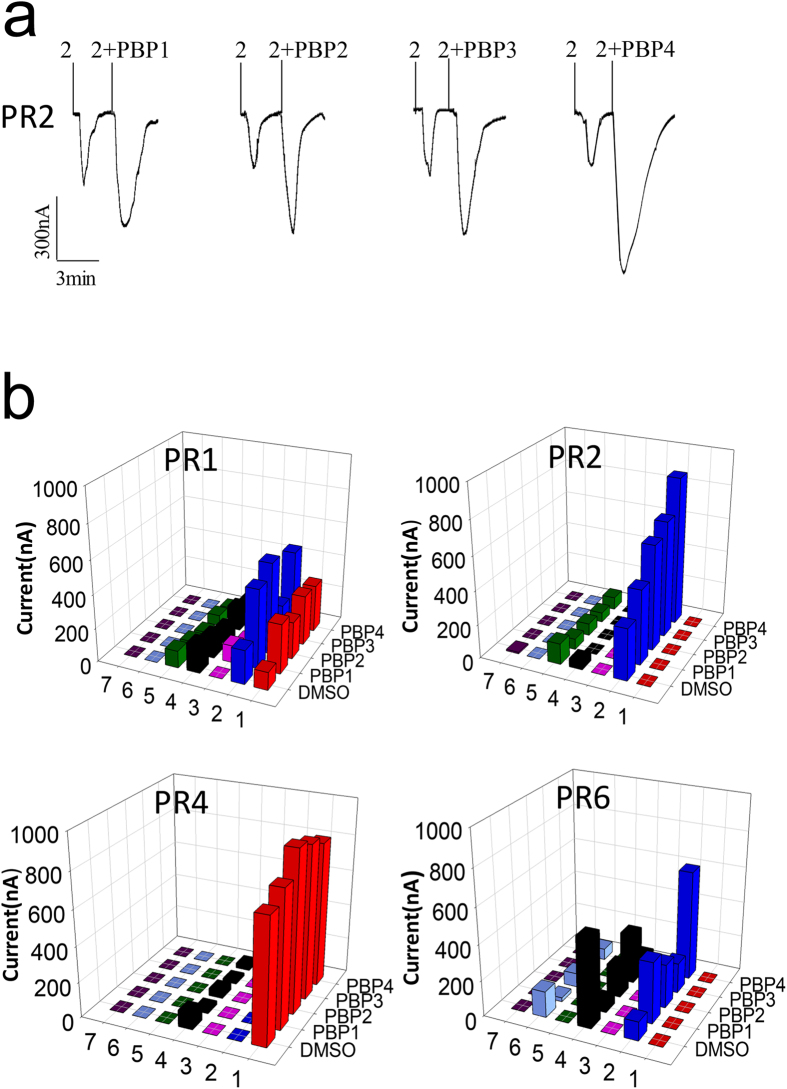
CsupPBPs can enhance the responses of CsupPRs to sex pheromone components. (**a**) Inward current response of CsupPR2/CsupOrco-coexpressed *Xenopus* oocytes to 10^−4^ mol/L sex pheromone. (**b**) Response values of CsupPRs/CsupOrco-coexpressed *Xenopus* oocytes to 10^−4^ mol/L ligands with either DMSO or CsupPBP. 1–7 are Z9–16:Ald, Z11–16:Ald, Z13–18:Ald, Z9–14:OH, Z11–16:OH, Z9,E12–14:Ac and 16:Ald. Experiments were performed in quadruplicates and average values are reported.

Having verified that four PRs respond to pheromone components and analogues with robust signals, when expressed in *Xenopus* oocytes, we asked how the presence of PBPs would affect such responses. In a first set of data we found that in general the addition of PBPs increases the intensity of the signal. To address the question of whether the effect observed could be only due to a better solubilisation of the hydrophobic ligands by the PBPs, we used DMSO as a carrier for the stimuli. This compound which is a good organic solvent and at the same time miscible with water and not affecting the performance of the proteins, was used at the same concentration in all experiments. The upper panel of Fig. 4 reports, as an example of four replications performed, the traces obtained when PR2 was stimulated with the major pheromone component Z11-16:Ald in the presence of each of the four PBPs. The lower panel summarises in graphical form all the data obtained with the four PRs that showed responses to the stimulants used. The effect of PBPs is not strictly specific nor completely aspecific with respect to the three components of the system: PR, PBP and ligand. For example, we can observe that with PR1 all PBPs produce similar positive effects on the responses of compounds 1 and 2. Instead, when studying the behaviour of PR6 in the presence of the four PBPs, the system seems to react in more specific ways. In fact, only the response of compound 2 is increased and mostly by PBP4. Therefore this receptor seems to be specific with respect to both pheromone and PBP. It is also interesting to observe that with this same PR6 the addition of all the PBPs produces a reduction of the signal obtained when the system is stimulated by compound 4. This effect could be explained by a trapping effect of the PBP, as observed in a recent work^34^. Rather than disproving the role of PBPs in enhancing the response of PRs to pheromones, this fact indicates that the presence of the correct PBP is necessary to obtain the effect, suggesting that specific interactions should take place between the three partners. Failure to establish such interactions may result in the trapping effect observed. Whether it is the complex PBP-ligand which is needed to activate the receptor or any other sort of interplay occurs between PR, PBP and ligand, we are not able to ascertain at the present state of knowledge. However, we can confidently assume that such interactions are not random, but regulated by the relative affinities of the three components for each other, even when the selectivity of the overall system seems rather poor.

### PBPs increase the sensitivity of PRs to pheromones

**Figure 5:**
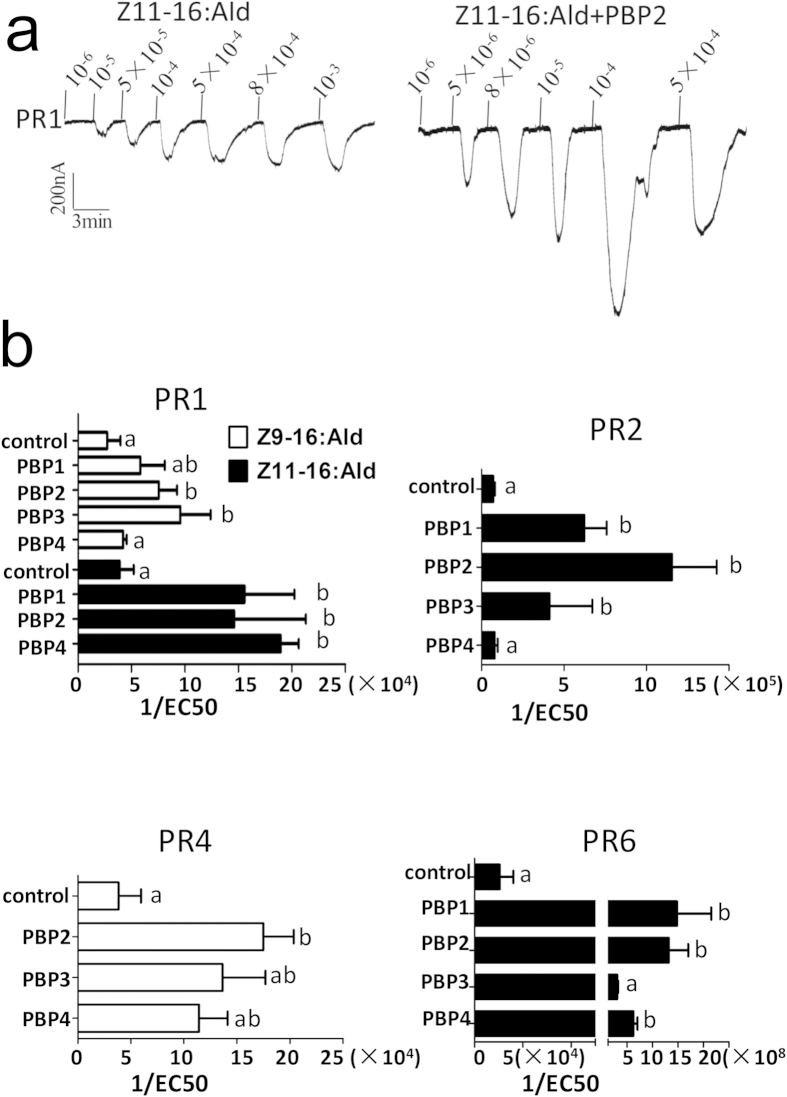
CsupPBPs can enhance the sensitivity of CsupPRs to sex pheromone components. (**a**) Dose-response of PR1 to Z11–16:Ald with or without PBP2. (**b**) Comparison of EC50 of CsupPRs to two sex pheromone components with and without CsupPBPs. The data were assessed by one-way analysis of variance (ANOVA). Error bars indicate SEM (n = 3 ~ 6)

In another series of experiments we tested the effect of PBPs on the sensitivity of PRs to the pheromone components by performing series of electrophysiological recordings using different concentrations of pheromones. The results, averages of four to five replications, are reported in Fig. 5 and enabled us to evaluate EC50 values for each experiment, that is for each PR/PBP pair. EC50 values are calculated from the dose/response curves and correspond to the flex point of each curve. These values represent a measure of the sensitivity, a sort of threshold for the response of each PR/PBP pair to single pheromone components. The upper panel of Fig. 5 reports representative examples of the actual traces recorded when using a concentration series of stimuli. The responses are dose-dependent in a regular fashion. The lower panel of the same figure contains graphical representations of the EC50 values, here reported as their reciprocals for a more immediate visualisation of the effects produced by the presence of PBP. Compared to the effect on the crude intensities of responses reported in Fig. 4, we can observe a much more dramatic consequence that PBPs produce on the system. In all cases we detected strong improvements in sensitivity, the most impressive being the case of PR6, whose sensitivity to the major pheromone component increases by four orders of magnitude in the presence of PBPs.

While in some cases all or most of the PBPs produce similar effects, in other combinations different behaviours have been observed in the presence of different PBPs. Typical is the response of PR2 to the major sex pheromone Z11-16:Ald. The sensitivity increases by more than one order of magnitude in the presence of PBP2 and to minor extents also in the presence of PBP1 and PBP3, while is not affected by PBP4. Again, these results indicate specific, although not exclusive, relationships between pheromones, PBPs and PRs.

While the results we have obtained in this work definitely show that PBPs play important roles in pheromone detection, they also indicate that a combinatorial code applies not only to the interactions between stimuli and receptors^46^, but also for what concerns the action of PBPs, both regarding their binding properties towards ligands and their interactions with receptors.

## Discussion

The main interest of our study was to investigate the role of PBPs in pheromone detection by monitoring their effects on the responses of PRs to pheromones. The results here reported can provide a contribution towards understanding the complex interplay between pheromones, receptors and binding proteins and the specific roles played by these three partners.

There is convincing evidence in the literature that OBPs are required for a correct functioning of the olfactory system in insects. In particular, the few reports available for studies of this type indicate that PBPs have a dual effect on the electrophysiological traces: (a) increase the absolute signals and (b) lower the threshold of response. Based on such observations, we first dissected the relationships between the four PBPs expressed in the antenna of *C. suppressalis* and their ligands through a series of binding assays where the affinity of each PBP was measured to the three pheromone components and other structurally related compounds. The picture emerging from this study shows for all four PBPs high affinity to pheromones and analogues, with dissociation constants well below micromolar concentrations, but very poor specificity in discriminating between individual semiochemicals. Variations in the affinities of each protein towards different ligands, as well as between PBPs, however significant, are too small when compared to the highly specific discrimination system of the moth’s antenna. These results, however, are not unexpected, being in agreement with similar studies reported in the literature for other moths^47^,^48^,^49^,^50^.

On the other hand, the key role of receptors in olfactory transduction is widely accepted and supported by a large number of studies^4^,^51^,^52^,^53^,^54^. Therefore, we measured the responses of the six PRs identified in the transcriptome of *C. suppressalis*^44^ using the *Xenopus* oocytes expression system, widely employed and accepted as a highly reliable tool. The three pheromone components and four selected analogues were used as stimuli for the six receptors. Compared to the PBPs, the responses of PRs were clearly differentiated, with two of them tuned mainly to the major pheromone component (Z9–16:Ald), two more responding also to other stimuli and the last two showing no response to any of the tested chemicals. In particular, the absence of activity observed with PR3 and PR5 may be due to several reasons: they might be tuned to pheromones of other species sharing the same environment with the *C. suppresalis* or else they may have lost their function during evolution in times not ancient enough to allow for mutations producing pseudogenes.

The response pattern of each of the four active PRs to closely related molecules, such as the pheromone components and their analogues, is differentiated enough to suggest that receptors alone could represent a robust array of sensors able to discriminate between different semiochemicals. However, the abundant presence of OBPs between the external environment and the receptors cannot be overlooked, as these are the proteins which in the natural environment first meet and make interactions with odorants and pheromones.

Therefore, once having monitored the binding of pheromones to PBPs *in vitro* and the response of PRs to the same stimuli, the main question we wanted to address was how PBPs could interfere with pheromones on their way to receptors and how the final signals could be affected by their presence. In general, we observed that the addition of PBPs to the *Xenopus* oocyte system produced stronger response signals. The effect is not due to a better solubilisation of the hydrophobic pheromones in the aqueous medium, as in all experiments the stimuli were delivered in DMSO. We also observed that the extent of such effect is often protein-dependent, with some PBPs enhancing the response of the same receptor for a specific pheromone more than others. This is, for instance, the case of PR6, which is more strongly activated by PBP4, when stimulated with the pheromone component Z11-16:Ald. Finally, for some combinations PR/PBP we observed an inhibitory effect relative to the signals recorded from the receptors alone. In such events we can hypothesise that the conformational change induced on the PBP by the ligand prevents interaction with the receptor (or alternatively delivery of the ligand to the receptors), resulting in a decreased concentration of the pheromone in the mixture. We observed this phenomenon with all four PRs when stimulated by Z9–14-OH (which is not a pheromone component of *C. suppressalis*) in the presence of each of the four PBPs. Such inhibitory effects, previously reported in the literature^34^, could indicate that either the PBP or the ligand (or both) did not match the receptor. From this point of view, if we assume that only the combination of a given receptor and a corresponding PBP could represent a functioning sensor for a given semiochemical, any other combination where one of the three elements does not match the others would result in a lack of response or in a reduction of the signal.

Concomitant to the increase in response, we also obsterved that the presence of a PBP often lowers the reaction threshold, making the system more sensitive. Particularly dramatic is the case of PR6, where all four PBPs increase the sensitivity to the major pheromone component by about three orders of magnitude. This observation, together with the fact that this receptor is more highly expressed in females than males, may suggest that it is tuned to other semiochemicals than those used in the present study. These might be sex pheromones from other species, whose presence the female should be aware of to avoid competion.

Putting together our observations, we can draw a picture of the olfactory system making use of two types of binding proteins to detect and differentiate between pheromones, PBPs and PRs, both broadly tuned and each lacking the high selectivity of the behavioural response of a moth to its own pheromone. When working in combination, PBPs and PRs produce a system endowed with improved sensitivity and in some cases more narrowly tuned. However, this emerging model is still far from a system using highly specific sensors each tuned to a single component of the pheromonal mixture.

The reason for having a broadly tuned set of detectors is to be related to the large number of stimuli challenging the moth’s antenna in the environment. In fact, besides the alluring call of the female of the same species, a male moth has to recognise and discard all the other signals produced by females of other species sharing the same environment. Moth pheromones are very similar in structure, often only differing by the position of a double bond. Besides, it is common the case of different species using the same molecules as components of their pheromonal blends.

To cope with such variety of stimuli and being able at the same time to appreciate differences in relative concentrations in the components of a mixture, a detection system equipped with broadly tuned sensors is more efficient and better adaptable than a rigid system using highly specific receptors.

How the combinations of 4 PBPs and 4 PRs can accomplish such tasks is still largely unknown. We know that PBPs are required for olfaction and we know that specific combinations of PRs and PBPs can produce highly sensitive detectors. However, how the PBPs can affect the performance of the receptors is a question far from being answered and we are not even in the position of buiding a reasonable model. The idea that the pheromone molecules produce a conformational change in the PBP, which in turn is sensed by the receptor is very appealing and has been suggested with at least two different models^35^,^55^. However, in both cases serious criticism has been raised towards such hypotheses^37^,^56^.

Studies like the present one provide information, which may eventually suggest better focused hypotheses and strategies to tackle one of the main basic question still unanswered in olfaction, the role of OBPs in odour coding.

## Methods

### Insect rearing and tissue collection

The striped rice stem borer *Chilo suppressalis* were reared at the Institute of Plant Protection, Chinese Academy of Agricultural Sciences, Beijing, China. Pupae were sexed before eclosion and kept separately in cages at 25 ± 1 °C, 60 ± 5% relative humidity with a photoperiod of 14:10 (light: dark). Tissues were collected from unmated 3-day-old moths and immediately frozen in liquid nitrogen.

### Expression profiles of PBPs and PRs

The relative expression levels of PBPs and PRs in male and female antenna were examined by qRT-PCR on ABI Prism 7500 (Applied Biosystems, Carlsbad, CA, USA), using the following qRT-PCR programme: 95 °C for 5 min; 40 cycles for 95 °C for 15 s, 60 °C for 1 min. A house-keeping gene, *CsupG3PDH* was used as a reference gene. All experiments were performed in duplicates. The relative expression quantities of PRs and PBPs were calculated using the comparative 2^−△△Ct^ method^57^. The sequences of all primers used in this assay are listed in supplementary Table S1.

### Bacterial expression and purification of CsupPBPs

The sequences of CsupPBPs encoding mature protein PBPs were amplified and cloned into PET30a vectors (Novagen, Madison, WI) which were used to transform BL21 (DE3) *E.coli* competent cells (Transgen). A selected positive clone was grown overnight and then used to inoculate 2 L liquid medium. After induction with 1 mM IPTG, the cell were grown for 8 h at 28 °C and harvested by centrifugation. After sonication of the pellet, recombinant proteins mainly existed in inclusion bodies. These were solubilised in binding buffer (8 M urea, 0.5 M NaCl, 5 mM Imidazole, 1 mM β-mercaptoethanol and 20 mM Tris–HCl pH 7.4) and purified by HisTrap affinity columns (GE Healthcare Biosciences, Uppsala, Sweden). Purified proteins were refolded by gradient dilution at 4 °C. After cleavage of the His-tag by treatment with recombinant enterokinase (rEK) (Novagen), PBPs were chromatographed again on HisTrap affinity columns and finally dialyzed against 50 mM Tris-HCl (pH = 7.4) overnight at 4 °C.

### Fluorescence binding assays

In order to measure the affinity of CsupPBP1–4 to the fluorescent probe N-phenyl-1-naphthylamine (1-NPN), a 2 μM solution of each CsupPBP in 50 mM Tris-HCl buffer was titrated with 1 mM 1-NPN in methanol to final concentrations of 2–16 μM. The affinities of CsupPBP1–4 to other ligands were measured in competitive binding assays, where a 2 μM solution of both CsupPBPs and 1-NPN was titrated with 1 mM methanol solutions of each lignd to final concentrations of 0.2–1.6 μM. The Prism software was used to analyse the data and plot the binding curves. The ability of ligand to compete with 1-NPN was measured in competitive binding assays, where a mixture of the protein and 1-NPN, both at the concentration of 2 μM were titrated with 0.1 mM or 1 mM solutions of each ligand to the final concentrations reported in the figures. Fluorescence intensities are reported as percent of the values measured in the absence of competitors. All experiments were performed in triplicates and mean values and standard errors are reported. Binding constants of competitors were calculated from the corresponding IC_50_ values using the equation: K_D _= [IC_50_]/1 + [1-NPN]/K_1-NPN,_ [1-NPN] being the free concentration of 1-NPN and K_1-NPN_ being the dissociation constant of the complex Protein/1-NPN.

### Receptor expression *in vitro* and electrophysiological recordings

The ORFs encoding *CsupPRs* were amplified and cloned into pT7Ts vector using specific primers (Supplementary Table S1). The cRNAs of all CsupPRs were synthesized using mMESSAGE Mmachine T7 kit (Ambion, Austin, TX).

The entire coding region of each CsupOR was sub-cloned into the XhoI/NotI sites of pT7Ts vector (Invitrogen) (Kozak sequence added before the cutting site in forward primer). The cRNAs of all CsupPRs were synthesized using mMESSAGE Mmachine T7 kit (Ambion, Austin, TX).

Electrophysiological recordings were performed according to previously reported protocols [31,58]. Mature healthy oocytes (stage V–VII) (Nasco, Salida, California) were treated with collagenase I(GIBCO, Carlsbad, CA) in washing buffer (96 mM NaCl, 2 mM KCl, 5 mM MgCl2, and 5 mM HEPES [pH = 7.6]) for about 1 h at room temperature. After being cultured overnight at 18 °C, oocytes were microinjected with 27.6 ng CsupORs cRNA and 27.6 ng CsupOrco cRNA. After injection, oocytes were incubated for 4–7 days at 18 °C in 1X Ringer’s solution (96 mM NaCl, 2 mM KCl, 5 mM MgCl2, 0.8 mM CaCl2, and 5 mM HEPES [pH = 7.6]) supplemented with 5% dialysed horse serum, 50 mg/ml tetracycline, 100 mg/ml streptomycin and 550 mg/ml sodium pyruvate.

Whole-cell currents were recorded from the injected *Xenopus* oocytes with a two-electrode voltage clamp. Odorant induced currents were recorded with an OC-725C oocyte clamp (Warner Instruments, Hamden, CT) at a holding potential of 280 mV. All experiments on PR responses were performed in six replicates, those involving interactions with PBPs in four replicates.

Data acquisition and analyses were carried out with Digidata 1440A and pCLAMP 10.2 software (Axon Instruments Inc., Union City, CA). Statistical comparison of responses of oocytes to the tested ligands and dose-response data were analysed by GraphPad Prism 5.0 (GraphPad Software Inc., San Diego, CA).

Tested pheromone components and analogs were purchased from Nimrod Inc (Changzhou, China, purify 96%). they were dissolved in dimethyl sulfoxide (DMSO) to 1 M Stock solutions and stored at −20 °C. Before testing, the stock solutions were diluted with 1 X Ringer’s buffer (96 mM NaCl, 2 mM KCl, 5 mM MgCl2, 0.8 mM CaCl2 and 5 mM HEPES [pH = 7.6]).

## Additional Information

**How to cite this article**: Chang, H. *et al.* Pheromone binding proteins enhance the sensitivity of olfactory receptors to sex pheromones in *Chilo suppressalis*. *Sci. Rep.* **5**, 13093; doi: 10.1038/srep13093 (2015).

## Supplementary Material

Supplementary Information
