# Supplementary material for: Pheromone binding proteins enhance the sensitivity of olfactory receptors to sex pheromones in Chilo suppressalis
Source: Sci Rep. 2015 Aug 27;5:13093. doi: 10.1038/srep13093 (PMC4550830; doi:10.1038/srep13093)
Supplement: Supplementary Information [file srep13093-s1.pdf]

Pheromone binding proteins enhance the sensitivity of olfactory receptors to sex pheromones in *Chilo suppressalis*

Hetan Chang[1,2], Yang Liu [1], Ting Yang [1], Paolo Pelosi [1], Shuanglin Dong [2,*], Guirong Wang [1,*]

Supplementary Table S1. Primers used in this study

Primer name	Sequence (5' – 3')
Primers for Real-time PCR	
CsupPBP1-F	GAGGGCACTGCTGGTATATTG
CsupPBP1-R	TGGTGGACAGGCAGAGTAT
CsupPBP2-F	GAAGTGGACACGGACTTCTTTA
CsupPBP2-R	GCTTGTTGGACATGCACATAAT
CsupPBP3-F	GCGACAGAGTCCGTGAAATAG
CsupPBP3-R	GTAGCCACGAGTTCGTTTATGA
CsupPBP4-F	CCAACTCTGGAAGGAGAACTATG
CsupPBP4-R	CCATGCTCGAATTTGCCTTG
CsupG3PDH-F	GTTGTGCCTCACCAATTTGTCAG
CsupG3PDH-R	GCCACCTTCAGCGATGTCG
CsupPR1-F	GCGTTGGCAAGCACTTATTT
CsupPR1-R	GTTATTGGTCCGTATTGTGCTAATG
CsupPR2-F	CCGACGACGTCTCTTCTTTATT
CsupPR2-R	GATCTAATTGGGAGCACTCCAG
CsupPR3-F	GTTACGGACCGCTTACAGTTAT
CsupPR3-R	CATACTCTCCCACGGCATTT
CsupPR4-F	TGGATGCCTCTTCTCTGTCTA
CsupPR4-R	CAGATGTCACGAGTTCCACTAC
CsupPR5-F	TTGGATGCCACTTACCTGTTTA
CsupPR5-R	CGGACTTAGATGTCACGAGTTC
CsupPR6-F	GTCCAGAGCCAGAAACTCATAG
CsupPR6-R	TGTAGCAAGAAGCACACAGAT
Primers for constructing expression vector	
CsupPBP1-F	CCG*GAATTC*TCACAAACGGTGATGAAATCAAT(*EcoRI*)

CsupPBP1-R	CCG*CTCGAG*TCAACGCATTTCTGCGATCA*(XhoI)*
CsupPBP2-F	CCG*GAATTC*TCTCAGGAGATCATGAAACAATTATC(*EcoRI*)
CsupPBP2-R	CCG*CTCGAG*TTATACGTCCGCCAGAACCTC(*XhoI*)
CsupPBP3-F	CCG*GAATTC*TCACAGGAACTCATAAAAAAGATGA(*EcoRI*)
CsupPBP3-R	CCG*CTCGAG*TCAAATTGCTTTGGTAGCCAC(*XhoI*)
CsupPBP4-F	CCG*GAATTC*AGGGAAGTTGAAATGGTACCTGA(*EcoRI*)
CsupPBP4-R	CCG*CTCGAG*TCAAGCTTCTGCTACAATCTCTTCTAC(*XhoI*)
CsupPR1-F	CCG*CTCGAG*gccaccATGGATTTTGAACTAAAAGAAAATAGATT(*XhoI*)
CsupPR1-R	AAGGAAAAAA*GCGGCCGC*TTAAACTGTTGATCGCAGAAAGG(*NotI*)
CsupPR2-F	CCG*CTCGAG*gccaccATGGATTCTGAACTAAAAGAAAATAGATT(*XhoI*)
CsupPR2-R	AAGGAAAAAA*GCGGCCGC*TTAAACTATTGATCGTAGAAAGGTGAAG(*NotI*)
CsupPR3-F	CCG*CTCGAG*gccaccATGAGCGGAGACACAAAAATAACTC(*XhoI*)
CsupPR3-R	AAGGAAAAAA*GCGGCCGC*CTATTCGTTTCTGGATTGCAGAAA(*NotI*)
CsupPR4-F	CCG*CTCGAG*gccaccATGTTCAACAATAAGGAGATTGAAAAT(*XhoI*)
CsupPR4-R	AAGGAAAAAA*GCGGCCGC*CTTAATCGTACTGCTGTTCGTTTTC(*NotI*)
CsupPR5-F	CCG*CTCGAG*gccaccATGTTCAATAAGTGGATTGAAAATGG(*XhoI*)
CsupPR5-R	AAGGAAAAAA*GCGGCCGC*TCAATCGGCGAAAGAACGAA(*NotI*)
CsupPR6-F	CCG*CTCGAG*gccaccATGGATTCTGCACCAAAAGAAAA(*XhoI*)
CsupPR6-R	AAGGAAAAAA*GCGGCCGC*TTAAACTATTGATCGTAGAAAGGTGAAA(*NotI*)

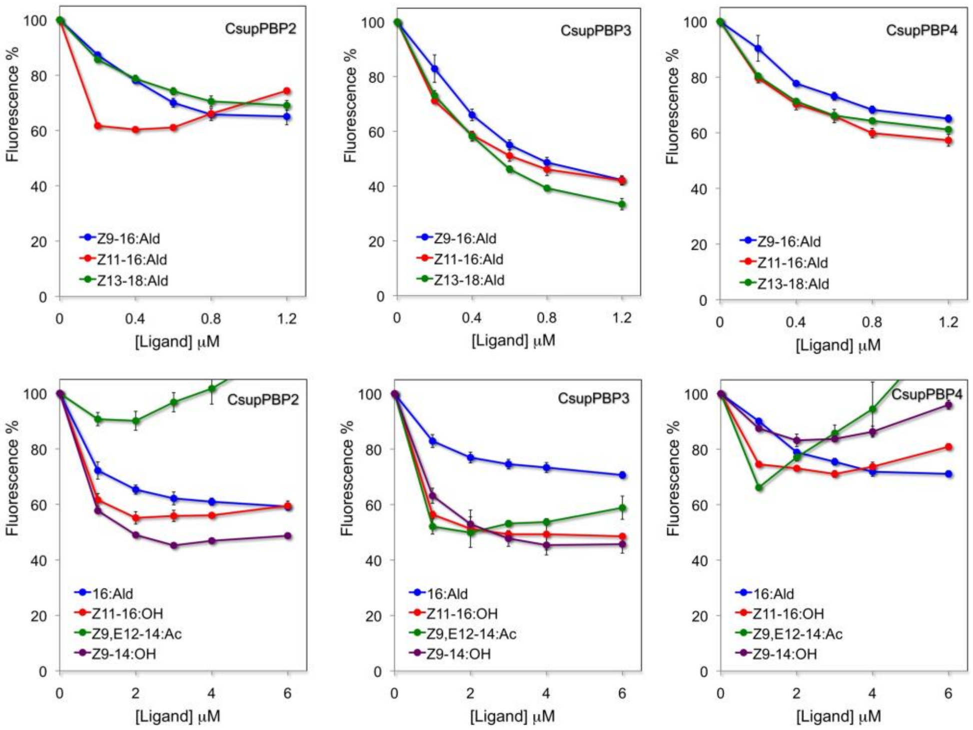

Figure S1. Displacement binding curves of 1-NPN from complexes with CsupPBP2, CsupPBP3 and CsupPBP4 by the ligands reported. Experiments were performed in triplicates and averages with SEM are plotted. Protein and 1-NPN, both at the concentration of 2 μM were incubated in 50 mM Tris and ligands were added as 1 mM or 0.1 mM solutions in methanol to the final concentrations reported in the graphs.
